# Metagenomics reveal unrestricted dispersal of extreme halophiles and higher connectivity among coastal vs. inland solar salterns and hypersaline lakes

**DOI:** 10.1093/ismeco/ycag165

**Published:** 2026-06-13

**Authors:** Tomeu Viver, Juan F Gago, Esteban Bustos-Caparros, Borja Aldeguer-Riquelme, Luis Miguel Rodriguez Rojas, Ana S Ramírez, Luciana Albuquerque, Souad Amiour, Aharon Oren, Mehmet Burcin Mutlu, Stephanus N Venter, Bonnie K Baxter, María E Llames, Bernardo González, Gustavo Rodríguez-Valdecantos, Horia L Banciu, Matthew B Stott, Fernando Santos, Brian P Hedlund, Josefa Antón, Rudolf Amann, Konstantinos T Konstantinidis, Ramon Rossello-Mora

**Affiliations:** Marine Microbiology Group, Department of Animal and Microbial Biodiversity, Mediterranean Institute for Advanced Studies (IMEDEA, CSIC-UIB), Esporles 07190, Spain; Marine Microbiology Group, Department of Animal and Microbial Biodiversity, Mediterranean Institute for Advanced Studies (IMEDEA, CSIC-UIB), Esporles 07190, Spain; Marine Microbiology Group, Department of Animal and Microbial Biodiversity, Mediterranean Institute for Advanced Studies (IMEDEA, CSIC-UIB), Esporles 07190, Spain; School of Civil & Environmental Engineering, Georgia Institute of Technology, Atlanta, GA 30332, United States; Department of Physiology, Genetics and Microbiology, University of Alicante, San Vicent del Raspeig, Alicante 03690, Spain; Department of Microbiology, and Digital Science Center (DiSC), Universität of Innsbruck, 6020 Innsbruck, Austria; Unidad de Epidemiología y Medicina Preventiva, IUSA, Facultad de Veterinaria, Universidad de Las Palmas de Gran Canaria, C/Trasmontaña s/n, Arucas, Canary Islands 35413, Spain; CNC-UC—Center for Neuroscience and Cell Biology and CIBB—Center for Innovative Biomedicine and Biotechnology, University of Coimbra, UC-Biotech, BiocantPark, Núcleo 04, Lote 8, Cantanhede 3060-197, Portugal; Laboratory of Bioengineering, National Higher School of Biotechnology, Ali Mendjeli University Town, E66 P.O. Box. Constantine 25100, Algeria; Department of Plant and Environmental Science, The Institute of Life Sciences, The Hebrew University of Jerusalem, The Edmond J. Safra Campus, Jerusalem 9190401, Israel; Department of Biology, Faculty of Science, Eskisehir Technical University, Eskisehir 26470, Turkey; Department of Biochemistry, Genetics and Microbiology, and Forestry and Agricultural Biotechnology Institute (FABI), University of Pretoria, Hatfield, 0028, Pretoria, South Africa; Great Salt Lake Institute, Westminster University, Salt Lake City, UT 84105, United States; Laboratorio de Ecología Microbiana Ambiental, Instituto Tecnológico de Chascomús (CONICET-UNSAM), Escuela de Bio y Nanotecnologías (UNSAM), B7130 Chascomús, Buenos Aires, Argentina; Facultad de Ingeniería y Ciencias, Universidad Adolfo Ibáñez–Center for Applied Ecology and Sustainability, Santiago de Chile 7941169, Chile; Facultad de Ingeniería y Ciencias, Universidad Adolfo Ibáñez–Center for Applied Ecology and Sustainability, Santiago de Chile 7941169, Chile; Department of Molecular Biology and Biotechnology, Faculty of Biology and Geology, Babeș-Bolyai University, 400006 Cluj-Napoca, Romania; Emil G. Racoviță Institute, Babeș-Bolyai University, 400006 Cluj-Napoca, Romania; School of Biological Sciences, University of Canterbury, 8041 Christchurch, New Zealand; Department of Physiology, Genetics and Microbiology, University of Alicante, San Vicent del Raspeig, Alicante 03690, Spain; School of Life Sciences, University of Nevada, 4505 S. Maryland Pkwy, Las Vegas, NV 89154-4004, United States; Department of Physiology, Genetics and Microbiology, University of Alicante, San Vicent del Raspeig, Alicante 03690, Spain; Department of Molecular Ecology, Max Planck Institute for Marine Microbiology, 28359 Bremen, Germany; School of Civil & Environmental Engineering, Georgia Institute of Technology, Atlanta, GA 30332, United States; Ocean Science & Engineering, School of Biological Sciences, Georgia Institute of Technology, Atlanta, GA 30332, United States; Marine Microbiology Group, Department of Animal and Microbial Biodiversity, Mediterranean Institute for Advanced Studies (IMEDEA, CSIC-UIB), Esporles 07190, Spain

**Keywords:** halophiles, distance decay patterns, genomics, metagenomics, ANI, biogeography

## Abstract

Hypersaline environments constitute ideal systems for studying evolutionary processes and microbial diversification due to their relatively low (and thus tractable) diversity and geographically isolated nature. Based on metagenomic sequencing of samples from 25 hypersaline sites in 11 countries taken within a single year, we explored the relationships between environmental factors, geographic distance, and microbial community structure and diversification. Our results revealed that microbial communities of coastal sites were more similar to each other than those of the inland sites, reflecting higher connectivity due to ocean currents and nearly unrestricted dispersal. Conversely, inland hypersaline environments showed less connectivity and higher genetic and taxonomic dissimilarities that did not correlate with the distance between the sampled sites. The latter results reflect reduced species migration characterizing inland sites as well as site-specific environmental factors selecting for divergent taxa. The 484 MAGs recovered, representing 284 distinct species, revealed a striking global ubiquity, with 62.5% of the species showing cosmopolitanism, defined as being present at both coastal and inland sites. Most cosmopolitan species showed allopatric differentiation, reflected by an increased frequency of non-synonymous substitutions between MAGs of the same species recovered from more distant sites. However, a few cases of truly cosmopolitan genomovars (average nucleotide identity, or ANI > 99.8%), were also observed. Our results suggest that extreme halophiles have nearly unrestricted global dispersal among ocean-connected sites, and to a lesser extent, among geographically isolated inland sites, although cases of allopatric diversification were also observed.

## Introduction

A longstanding challenge in microbial ecology is to identify the forces shaping microbial biogeography and the drivers of species dispersal and community assembly [1, 2]. Classical niche-assembly theory, as introduced by the Baas–Becking principle, proposes that microbes disperse widely, with environmental conditions determining local community composition [3]. In contrast, distance–decay patterns and neutral theory highlight dispersal limitation, stochastic drift, and homogenization constrained by geographic or environmental gradients [4, 5]. Evidence of evolutionary independence among geographically isolated populations challenge the “everything is everywhere” principle [6], and environmental changes, including geological events, climatic change and/or long-term habitat isolation, may explain deviations from distance–decay patterns [4]. Accordingly, there have been very few examples of truly cosmopolitan species, defined as genomovars that are identical among distant sites and/or intra-species diversity that does not correlate to geographic distance, and these are mostly from the ocean environment [7]. It remains largely unclear if such cosmopolitan taxa can be found within more isolated environments such as hypersaline lakes, a gap that our sampling aimed to address (see also below). Microorganisms could overcome isolation barriers by persisting in dormant states [8, 9], and disperse via ocean currents [10], clouds and dust [11–13], and migratory animals [14, 15]. However, within isolated environments, microbes are thought to be predominantly subjected to dispersal limitations, genetic bottlenecks, and/or heterogeneous selection caused by environmental factors, leading to allopatric diversification, and thus speciation, being the major evolutionary process [16–19].

Hypersaline systems harbor stable and highly similar microbial communities across the globe [20–22], dominated by the archaeal class *Halobacteria* and the bacterial family *Salinibacteraceae* [23–26]. Solar salterns differ from naturally occurring hypersaline lakes by their semiartificial nature (i.e. human-controlled salt concentrations by directly feeding the ponds with seawater) that promotes stability (e.g. buffering against precipitation-driven dilution or fluctuating aridity that cause desiccation). Given that solar salterns are human-engineered environments, they are relatively recent habitats for extreme halophiles. Some of these environments have over 2500 years of history, such as the Salterns of S’Avall, which date back to the Punic colonization of Mallorca, Spain [27], whereas others are much younger, such as the salterns of Eilat in Israel that are only ~70 years old. Relatively young salterns also include inland solar salterns that are fed by groundwater dissolution of diapiric bedrock, such as the Soutpan salterns in South Africa, dated to 1880, or the Rio Maior salterns in Portugal, dated to 1177 (geological context provided in Table S1). It is worth noting that the brines used to feed the salterns may contain microorganisms released from the diapiric rocks [28, 29], which are substantially older. For example, diapirs associated with Rio Maior date to the Jurassic Period (201 to 145 million years ago) [30]. Natural saline lakes can also have older origins. For example, the Great Salt Lake in the USA is a remnant of Pleistocene Lake Bonneville (dated, at maximum, 30 000 years ago). As we previously hypothesized [31], the communities in such inland hypersaline sites might partially originate from trapped organisms within these sites or the feeding diapirs together with the aerial immigration of allochthonous extreme halophiles [11–15]. It also seems that human-managed salterns tend to maintain stable but less diverse communities than natural lakes [21, 32].

Despite the relatively low diversity typically observed in hypersaline environments at higher taxonomic ranks (e.g. family, order), these habitats typically exhibit considerable species and intra-species richness. For instance, *Salinibacter ruber* (*Sal. ruber*) may contain between 5000 and 11 000 distinct genomovars (defined as clusters of genomes sharing >99.5% ANI and >90% of their gene content [33, 34]) within a single pond [35–37]. In contrast, the archaeal species *Haloquadratum walsbyi* (*Hqr. walsbyi*) exhibits a remarkable homogeneity worldwide with only four genomovars identified to date [38]. Most extreme halophiles cannot grow with salt concentrations below 15% [39]; thus, dispersal through air or water should be as dormant cells [8], except via animals (e.g. seabird nostrils) that could transport active cells [14]. These characteristics make extreme halophiles both interesting and tractable systems for biogeography studies, as evolution occurs within discrete habitats with minimal expected evolution during dispersal and minimal establishment of new, allochthonous species.

Here, we applied community-level metagenomics, ion profiles and geographic distances to characterize both community structures and intraspecies diversity patterns of 25 coastal and inland sites within 11 countries, which represents an unprecedented dataset for these habitats. We specifically tested the hypothesis that coastal microbial communities are more similar to each other than their more isolated inland counterparts due to the homogenization caused by ocean currents.

## Material and methods

### Samples: Origin and processing

In 2018, an international research consortium formed the Halophile Sequencing Project (HSP) [20], involving 19 researchers from 11 countries. The HSP team collected samples from 25 hypersaline sites in these countries (Table 1). All samples were thalassohaline, i.e. with a direct (seawater) or indirect (dissolution of diapiric rocks) ocean water origin. For each sample, at least 1 L of brine was collected and used for the current metagenomic study. All samples were collected between September 2018 and January 2020, and were close to saturation levels for NaCl (25%–37% total salts).

**Table 1:** Sampling location, date and salinity of the hypersaline sites in study. Salinity (%) given as measured using a Sper Scientific Salt Refractometer.

Country	Location		Abbreviation	Hypersaline system	Sampling date	Coordinates	Salinity (%)
Spain	Mallorca	S’Avall	ES_AV	Coastal solar saltern	September 2018	39°19′28″N—2°59’21″E	36.2
		Es Trenc	ES_CM	Coastal solar saltern	September 2018	39°21′03″N—3°00’45″E	34
		Coco 1—Mallorca	ES_CO1	Coastal pool	May 2019	39°15′55″N—3°03′05″E	31
		Coco 2—Mallorca	ES_CO2	Coastal pool	May 2019	39°15′55″N—3°03′05″E	30
	Alicante	Santa Pola	ES_SP	Coastal solar saltern	May 2019	38°11′05″N—00°35′08"W	37
	Canary Islands	Janubio—Lanzarote	ES_LZ	Coastal solar saltern	June 2019	28°55′11″N—13°49′15"W	34.4
		Del Carmen—Fuerteventura	ES_FV	Coastal solar saltern	July 2019	28°21′58″N—13°52′12"W	35
		Arinaga 1—Gran Canaria	ES_CA1	Coastal solar saltern	October 2018	27°51′05″N—15°24′07"W	36
		Arinaga 2—Gran Canaria	ES_CA2	Coastal solar saltern	October 2018	27°51′05″N—15°24′07"W	34
Algeria	Oum El Bouaghi	Sebkha Ezzemoul	DZ_AGR	Inland lake	January 2020	35°53′13″N—6°30′50″E	30
Israel	Eilat	Eilat 3	IL_EL3	Coastal solar saltern	September 2018	29°33′29″N—34°57′54″E	33
		Eilat 6	IL_EL6	Coastal solar saltern	September 2018	29°33′29″N—34°57′54″E	34
Portugal	Rio Maior	Rio Maior	PT_RMP	Inland solar saltern	June 2019	39°19′34″N—08°55′17"W	32
Romania	Transylvania	Fără Fund	RO_FF	Inland lake	April 2019	45°52′34″N—24°04′03″E	29
Turkey	Konya	Tuz Lake 1	TR_TUZ1	Inland lake	June 2019	29°33′29″N—34°57′54″E	33
		Tuz Lake 2	TR_TUZ2	Inland lake	June 2019	29°33′29″N—34°57′54″E	ND
USA	Utah	Great Salt Lake	US_CM	Inland solar saltern	July 2019	40°41′13″N—112°22′33"W	37
Chile	Boyeruca	Lo Valdivia 1	CL_LV1	Coastal solar saltern	January 2019	34°41′56"S—72°00′44"O	26
		Lo Valdivia 2	CL_LV2	Coastal solar saltern	January 2019	34°41′56"S—72°00′44"O	26
Argentina	La Pampa	Guatraché	AR_G	Inland lake	July 2019	37°42′59"S—63°31′04"W	33
		Colorada Chica	AR_CCH	Inland lake	August 2019	38°22′51"S—63°25′41"O	33
		Colorada Grande	AR_CG	Inland lake	August 2019	38°15′41"S—63°45′02"O	32
New Zealand	Marlborough	Lake Grassmere	NZ_LG	Coastal lake	January 2019	41°44′33"S—174°08′35″E	37
South Africa	Western Cape	Velddrif	ZA_VC	Coastal solar saltern	January 2019	32°44′03"S—18°11′60″E	34
	Bloemfontein	Soutpan	ZA_BS	Inland solar saltern	January 2019	28°45′21"S—26°02′55″E	25

### Ionic composition, metagenome analyses, MAG reconstruction and intra-species comparisons

Detailed materials, methods, and statistical analyses are provided in Supplementary Text S1. Briefly, ionic composition of the samples was quantified by ion chromatography using a Metrohm, 850 ProfIC AnCat—MCS system. Similarity in ionic composition across the samples was assessed by PCA (scaled data) and non-metric multidimensional scaling (NMDS) (Bray–Curtis; using vegan v2.6 [40]).

DNA extraction was performed as detailed in Urdiain *et al.* [41]. Metagenomes were sequenced on an Illumina NextSeq500 (2 × 150 bp). Reads were trimmed using Bbtools with default settings. Fraction of the total diversity of the sample recovered by sequencing (or just coverage) and alpha diversity were estimated with Nonpareil v3.303 [42]. Beta diversity was calculated using Mash v2.2.2 [43] and visualized by NMDS (vegan v2.6 [40]). Correlations with geographic distance and ion concentration were assessed by Procrustes analysis [44].

Trimmed reads were assembled with SPAdes v3.13.1 (metaSPAdes) [45] and MEGAHIT v1.2.9 [46]. Genes were predicted with Prodigal v2.6.3 [47] from contigs >500 bp. Proteins were compared using DIAMOND v0.9.31 [48], and reciprocal best matches identified with the *rbm.rb* script [49] (≥50% identity over ≥50% length). Orthologous groups (OGs) were defined with *ogs.mcl.rb* [49], and their frequencies (coastal vs. inland metagenomes) were estimated with *Table.prefScore.R* [49]. OGs were annotated using COGclassifier v1.0.5 (https://github.com/moshi4/COGclassifier). AAI-based similarity was visualized by NMDS (vegan [40], 50%–90% identity thresholds).

Contigs >2 kb were binned with MaxBin v2.1.1 [50] and MetaBAT v2 [51]. MAG quality was assessed with CheckM2 v0.1.3 [52], retaining MAGs with >50% completeness and <10% contamination. MAGs were dereplicated with dRep v3.4.0 [53] at 96% ANI as a proxy for species, and re-clustered (FastANI [54], ≥20% overlap [55]) to obtain one representative per resulting cluster (or species). Taxonomy was assigned with GTDB-Tk v2.4 (GTDB R220) [56]. MAG abundances were estimated by mapping reads (Bowtie2; bedtools [57, 58]) and calculating TAD80 (*BedGraph.tad.rb* [49]), normalized by genome equivalents (MicrobeCensus v1.1.0 [59]). Species presence required non-zero TAD80 and ≥20% breadth. Species preference across samples was assessed with *Table.prefScore.R* [49]. Preference scores quantify the relative enrichment of each MAG across environments by comparing its observed frequency across samples to the expected frequency given its overall occurrence and the distribution of MAGs per sample, thus normalizing for uneven detection and sampling effort.

For intra-species MAG comparisons, ANI and shared genome fraction were calculated using the *ani.rb* script [49]. Intraspecies pangenomes were analyzed as in Conrad *et al.* [60], clustering genes into OGs with MMseqs2 v13.45111 [61] (90% identity, ≥70% coverage). Single-copy core genes were aligned with MUSCLE v3.8.31 [62], concatenated (*Aln.cat.rb* [49]), and used for phylogeny with RAxML in ARB v6.0.6 [63]. Single nucleotide polymorphisms (SNPs) were identified with Mauve [64], and classified as synonymous or non-synonymous using a custom Python pipeline. ANIr was estimated by mapping reads with Magic-BLAST+ v2.12.0 [65] (>95% identity).

## Results

### Geographic distribution of the samples and their ionic composition

The 25 brines were grouped into coastal or inland sites according to their water source origin (Table 1, S1 and Fig. S1). The 15 coastal hypersaline environments originated from the direct concentration of seawater. The nine inland hypersaline sites originated from the dissolution of subsurface halite deposits of different geological times (Table S1), and are either fed by groundwater brines via human activity or occur naturally. In addition, we collected brines from two ephemeral coastal ponds or “Cocos”, which are small ponds adjacent to the seashore that are seasonally filled with seawater by sea spray and waves breaking on the shore (Fig. S2).

**Figure 1:**
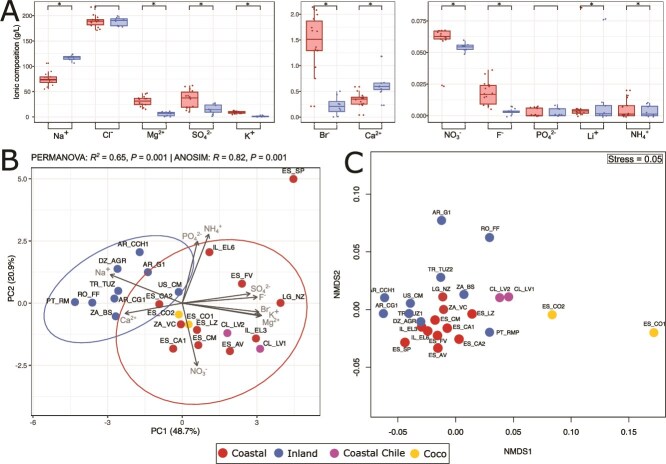
Similarity among all samples determined in this study. (A) Comparison of ionic composition (g/L) between coastal (red) and inland (blue) samples. (B) Principal component analysis (PCA) based on ionic composition. (C) NMDS analysis of Mash-based distances (beta diversity index). Each point on the plot represents an individual sample. An asterisk (*) marks statistically significant differences determined by the Wilcoxon test (*P* < .05).

All ionic compositions were predominantly composed of sodium salts, primarily NaCl (Fig. 1A and Table S1). Due to their seawater origin, coastal brines and Coco samples also contained a significant proportion of magnesium. In contrast, magnesium concentrations were four to five times lower in the inland sites, mirroring the composition of the rock that serves as the source of the dissolved salts, with strong predominance of halite (NaCl) and higher concentrations of calcium and lithium. In addition, we observed higher concentrations of nitrogen compounds (i.e. NO_3_^−^ and NH_4_^+^) in the coastal sites, but not phosphate. Ordinations using PCoA based on ionic composition (Fig. 1B) revealed a clear separation between coastal and inland environments, supported by a significant difference based on the ANOSIM test (*R* = 0.82, *P* = .001). PERMANOVA analysis indicated that 65% of the variation in ionic composition could be explained by the origin of the salt water (coastal vs inland) feeding the hypersaline sites. However, the Mantel test assessing the relationship between geographic distance and ionic composition did not show any significant correlation when coastal and inland sites were analyzed separately (*P* > .5; Table S2).

### Alpha and beta diversity measures based on whole metagenome comparisons

Metagenome sizes ranged from 5.2 to 15 Gbp (average 11.5 ± 2.3) after trimming, except for the Santa Pola (ES_SP) dataset, which was sequenced at 60 Gbp (Table S3). As estimated by Nonpareil, all datasets captured 89%–98% of the total microbial community, defined here as the fraction of community diversity recovered by the metagenomic reads (Fig. S3A). Alpha diversity comparisons using the Nonpareil sequence diversity index (*N_d_*, which is based on the level of redundancy of reads in each metagenome) revealed that the coastal solar salterns exhibited lower diversity (17.6 ± 0.9) than the inland hypersaline lakes (18.2 ± 0.6; note that *N_d_* is in natural log scale), which was statistically significant (Wilcoxon *P* < .05) (Fig. S3B). Inland samples, such as of the Tuz Lake, reached *N_d_* values up to 18.9.

Beta diversity, evaluated using Mash distance derived from reciprocal overall read-based k-mer sharing (Table S4), revealed a significant difference between coastal and inland sites (PERMANOVA analysis; *P* = .016). This difference remained significant (*P* = .032) after excluding the two most distant inland samples, AR_G1 (Argentina) and RO_FF (Romania). Moreover, coastal sites tended to share greater genetic similarity among themselves compared their inland counterparts, which displayed higher within-group variability. Betadisper analysis confirmed these patterns, with average distances to centroid of 0.053 for coastal and 0.075 for inland samples (Fig. 1C). An ANOVA test indicated that these differences in dispersion were statistically significant (F = 7.96, *P* = .01). When coastal and inland brines were analyzed separately, we did not detect any significant relationship between microbial community composition (based on Mash distances) and ionic composition, based on distance-based redundancy analysis (permutation test, *P* > .7), and Mantel tests (*P* > .4; Table S2). In these analyses, the Chilean and Coco samples were distant from the centroid (Fig. 1C). Thus, these samples are treated separately below as their different community compositions seem to be related to their different degree of community maturity, which we discuss later.

A total of 673 723 OGs were recovered by all metagenome assemblies, and the comparison of their occurrence between coastal and inland sites showed that 16.3% of OGs were more frequently found in coastal sites, 14.4% were more common in inland sites, and 69.2% showed no clear habitat preference (Fig. S4A and B). Among all detected OGs, 51.33% (*n* = 345 814 OGs) were assigned to a functional COG category. Despite the existence of inland- or coastal-enriched OGs, we did not find any special COG functional enrichment by sample type (inland vs coastal) (Fig. S4C).

### Recovered MAGs and their taxonomic diversity and ubiquity

The 25 metagenomes yielded 484 good-quality MAGs, with an average estimated completeness of 76.25% (inter-quartile range [IQR]: 65.5%–86.4%), and an average estimated contamination of 2.43% (IQR: 0.45%–3.56%). After dereplicating MAGs into species based on an ANI value threshold of >96% [66], we identified a total of 284 unique species (Table S5 and S6). GTDB-Tk [55] assigned 231 MAGs to the *Archaea* and 53 to the *Bacteria* domains. Among the representative MAGs of the 284 species, 67 MAGs were of high-quality [67], with >90% completeness (average 95 ± 2.8%) and <5% contamination (average 1.3 ± 1.34%). The most prevalent archaeal families were *Haloferacaceae* (*n* = 97 species), *Haloarculaceae* (*n* = 74), and *Nanosalinaceae* (*n* = 49), underscoring the dominance of members of the class *Halobacteria* in hypersaline environments. Among these, 80.1% could be identified to the genus level, with *Halorubrum* (*n* = 31), *Halovenus* (*n* = 28)*, Nanosalina* (*n* = 19), *Halobellus* (*n* = 17), and *Natronomonas* (*n* = 14) being the most species-rich genera (Table S6). Regarding bacterial species, 66% belonged to known genera and the most species-rich genera were *Salinibacter* (*n* = 11), followed by *Roseovarius* (*n* = 6), and *Spiribacter* (*n* = 5). Moreover, representatives of unclassified higher taxa were identified among the 284 MAGs within the classes *Bacteroidia* (*n* = 7), *Bradymonadia* (*n* = 3), and *Verrucomicrobiia* (*n* = 2).

To evaluate the presence and abundance of the retrieved species, metagenomic read recruitment against the 284 MAGs was used. The sum of reads recruited to these MAGs accounted, on average, for 61.3% (IQR: 56.4%–74.4%) of the total metagenomic reads. The sample from Lanzarote (ES_LZ) showed the lowest read recruitment (24.9%), whereas the sample from Algeria (DZ_AGR) the highest (87%) (Table S6 and Fig. S5).

**Figure 2:**
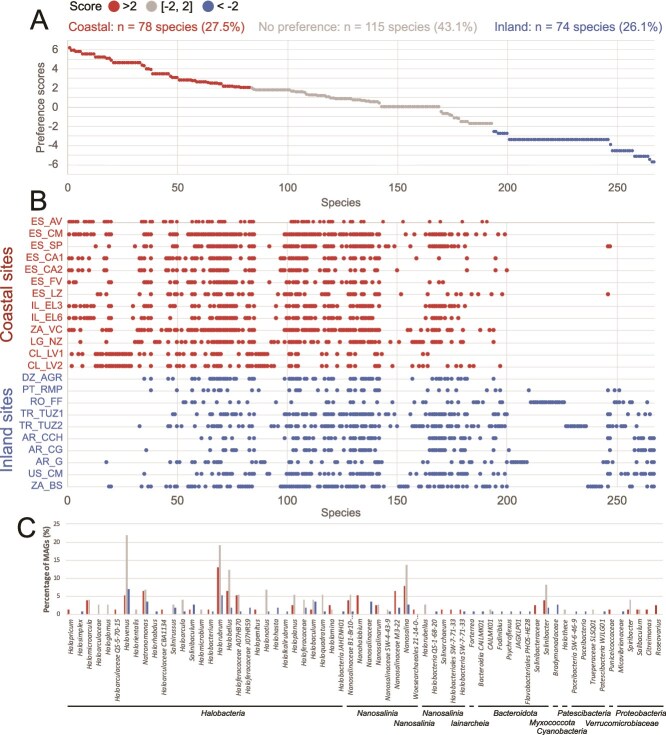
Preference scores of the recovered species in coastal and inland datasets. (A) Preference scores >2 indicate stronger preference for coastal samples while scores <2 reflect preference for inland samples. Each point represents a species, and the horizontal position corresponds to the same species as shown in panel B. (B) Each point indicates the presence or absence of each species in the coastal or inland samples included in this study and underlies the summary statistics shown in panel A. (C) Fraction of MAGs within each genus detected with preference for coastal, inland, or no preference.

As we did for the orthologous gene distribution, we analyzed the preference scores for the detected species between the coastal and inland sites (Fig. 2A and B and Table S7). We observed a remarkable ubiquity among most species, with 62.5% being detected in both coastal and inland hypersaline systems. Nonetheless, 78 species (27.5% of total) showed a clear preference for coastal sites (with 35 being exclusively coastal), and 74 (26.1%) showed a preference for inland samples (with 65 only detected at inland sites). A total of 115 species (43.1%) did not exhibit any preference for either environment. The most ubiquitous species present in both coastal and inland sites, in decreasing occurrence (Table S7), were *Halonotius* (*Hns.*) *pteroides* (23 sites), *Halorubrum* sp. sp_141_2 (22 sites), *Halonotius* sp. sp_89_2 (21 sites), *Sal. ruber* (20 sites), *Hqr. walsbyi* (18 sites), and *Haloquadratum* sp. sp_19_1 (17 sites). Several archaeal genera were exclusive to coastal sites, including *Halapricum, Halopenitus, Nanohalobium, Salinarchaeum* and *Haloplanus*, whereas *Halorhabdus, Halohasta*, and *Halalkalirubrum* were restricted to inland sites. In contrast, inland sites showed a relative enrichment of bacterial taxa, such as *Psychroflexus, Fodinibius*, and *Candidatus* Paceibacteria, as well as members of the families *Bradymonadaceae* and *Micavibrionaceae* (Fig. 2C).

### Intraspecific similarity patterns with geographic distance

**Figure 3:**
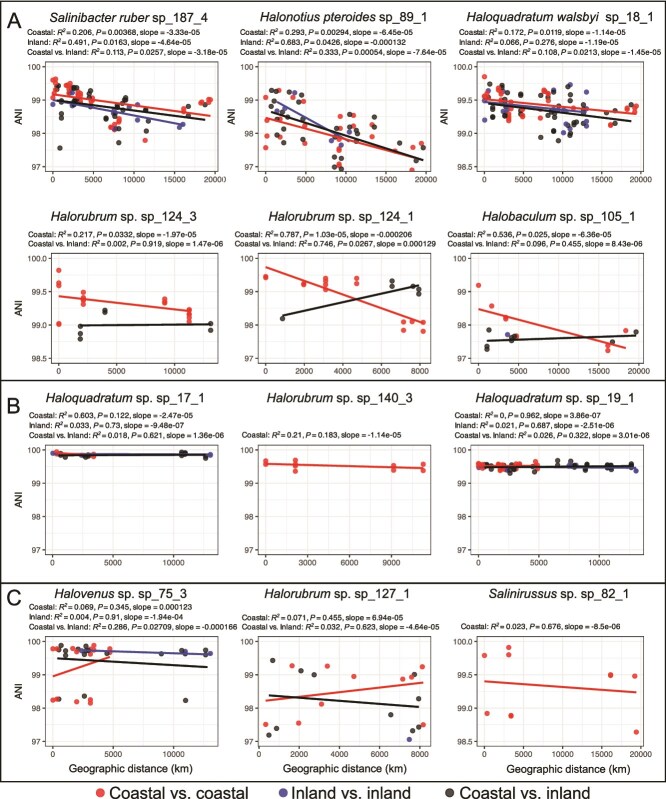
Relationships between intra-species average nucleotide identity (ANI) and geographic distances. The comparisons are based on pairwise ANI analyses between MAGs assigned to the same species. (A) Species showing an ANI (y-axes) decrease with increasing geographic distance (x-axes). (B) Highly conserved species with ANI values >99. (C) Species with no correlation between ANI and geographic distance.

**Figure 4:**
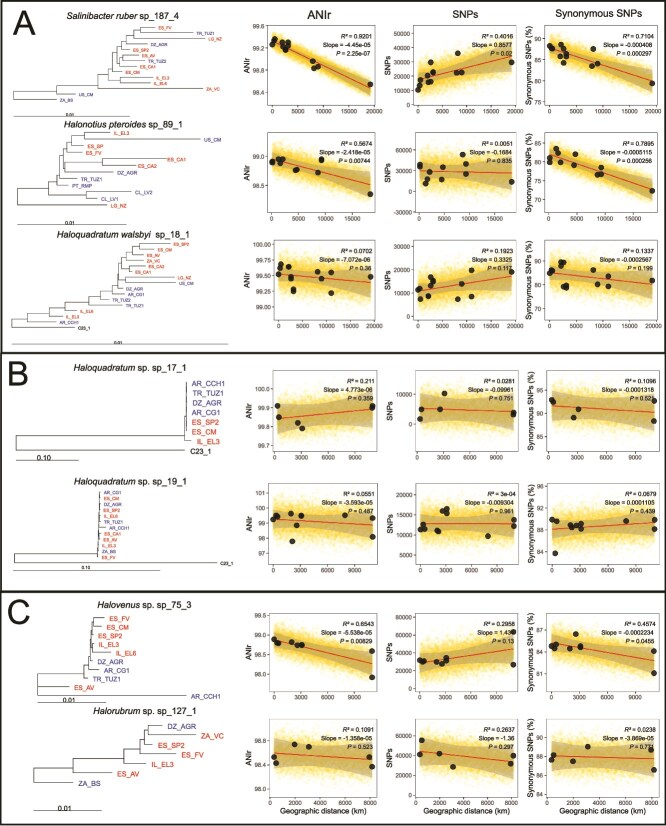
Comparison of the relationship between ANI and geographic distance with patterns observed in the core-gene phylogenetic tree, ANIr, number of SNPs, and the proportion of synonymous SNPs across increasing geographic distances. This figure shows a representative species for each of the three observed patterns. The full set of species is presented in Fig. S10. (A) Species showing a decrease in ANI (y-axes) with increasing geographic distance (x-axes). (B) Highly conserved species with ANI values >99. (C) Species with no correlation between ANI and geographic distance. The outgroups in the phylogenetic trees were selected from MAGs of closely related species or genera within our collection.

Twenty-two species were selected that each had a representative MAG recovered from at least 4 of the 25 metagenomes to evaluate correlations between geographic distance and genomic identity. The 22 selected species accounted for an average of 51 ± 19.4% of the total community in coastal sites and 32 ± 17.6% in inland sites (Fig. S6), thus representing the most abundant taxa. We observed three distinct patterns of genome similarities based on ANI values (Figs 3, 4, and  S7). The first pattern was a distance–decay relationship, whereby decreasing ANI values correlated with increasing distance. This pattern was found in 8 of the 22 species. Among these, two species (*Sal. ruber* sp_187_4 represented by 14 MAGs, and *Hns. pteroides* sp_89_1 represented by 11 MAGs) showed a significant ANI decline with distance regardless of their coastal or inland origin (*P* < .05; Fig. 3A). In contrast, the remaining six species (*Hqr. walsbyi* represented by 15 MAGs, *Halorubrum* sp_124_3 by 8 MAGs, *Halorubrum* sp_124_1 by 7 MAGs, *Halorubrum* sp_139_1 by 5 MAGs, *Halobaculum* sp_105_1 by 7 MAGs, and *Halobellus* sp_157_1 by 6 MAGs) showed the declining trend only in comparisons among coastal samples (Fig. 3A). For the three species with the highest number of binned MAGs (≥ 11: *Sal. ruber, Hns. pteroides,* and *Hqr. walsbyi*) the lowest ANI values were observed between coastal and inland MAGs, and the patterns were consistent for both ANI values calculated using the whole MAG sequence or only their orthologous core genes (Figs S7A and S8A, respectively). These results also revealed the stronger conservation of core gene sequences at short-range distance (from 0 to 400 km) relative to larger distances (Fig. S9A). To further corroborate these results, we examined the ANIr (i.e. Average Nucleotide Identity of reads) of metagenomic reads mapped against the reference MAGs. We observed a significant decrease of ANIr with increasing geographic distance between the sample that the reference MAG originated and the sample that the metagenomic reads originated from, for both *Sal. ruber* and *Hns. pteroides*, reflecting a corresponding increase in the number of SNPs and a statistically significant reduction in the proportion of synonymous vs. non-synonymous substitutions (*P* < .05; Figs 4A and S10A). Consistent with these patterns, intra-species phylogenies tended to reveal a higher phylogenetic similarity among coastal MAGs compared to inland MAGs (Figs 4 and S10). The coastal MAGs always clustered within the same phylogenetic branch, whereas the inland either affiliated in distinct branches or exhibited longer branch lengths. For *Sal. ruber*, inland MAGs generally showed greater phylogenetic divergence, with the most dissimilar MAGs recovered from inland sites in the USA and South Africa. For *Hns. pteroides*, the most phylogenetically divergent MAGs originated from New Zealand and the Chilean coastal sites. The correlation between ANI and geographic distance was clear even when the analysis was restricted to MAGs belonging to the same *Hqr. walsbyi* genomovar, with the most distant comparisons involving the coastal MAG from New Zealand and inland MAGs from the USA, Algeria, and Colorada Grande (Argentina) and showing an increase in divergence between these MAGs, supported by comparisons of ANI versus geographic distance, ANIr, and phylogenetic reconstructions (Fig. S10A). The five additional archaeal species affiliated with the genera *Halorubrum* (sp_124_3, sp_124_1, and sp_139_1), *Halobaculum* (sp_105_1), and *Halobellus* (sp_157_1), showed consistent patterns of decreasing genomic relatedness with increasing geographic distance only when comparing coastal MAGs. These patterns were evident across multiple genomic analyses, including ANI (Figs 3A and S7A), ANIr (Fig. 4A), and core gene similarity (Figs S8A and S9A). Consistently, phylogenetic analysis based on core genes revealed that inland MAGs formed distinct and phylogenetically distant clades relative to coastal MAGs (Fig. S10A).

A second pattern observed was MAGs with high ANI values regardless of geographic distance, indicating a high genome conservation across sampling sites. Four of the 22 species showed ANI values >99.5% (i.e. being represented by a single genomovar) while two species showed slightly lower ANI values (>99.0%, probably being represented by more than one, but closely related genomovars; Figs 3B and S7B). The lowest intra-species genome diversity observed was for *Haloquadratum* sp_17_1, with an ANI value of 99.85% ± 0.05%, on average, and a minimum of 99.75%. This highly conserved species was binned from metagenomes from distant salterns from the Mediterranean, Eilat, and New Zealand, and also from inland lakes in Algeria, Turkey, and Argentina. Similarly, the species *Haloquadratum* sp_19_1, which was recovered from 12 different sites, including both coastal (Canary Islands, Mallorca, Santa Pola, and Eilat) and inland sites (Algeria, Turkey, South Africa, and Argentina), exhibited an average ANI value of 99.5% ± 0.07% and a minimum of 99.3%, and no significant trend in ANI values or SNPs with distance based on core genes (Figs S9B, 4B and S10B). Altogether, the same or very closely related genomovars are present even at disparate sites suggesting high global dispersal rates.

Finally, a third pattern was observed in which there was no correlation between genome relatedness and geographic distance. Eight of the 22 species did not show correlation between distance and ANI (Figs 3C and S7C) or core genes (Fig. S9C). For seven out of these species, we detected highly conserved MAGs (ANI > 99.5%; same genomovar) among some geographically distant sites, but also MAGs with <99% ANI between close locations (i.e. distinct genomovars). For example, *Halovenus* sp_75_3 MAGs from Es Trenc (Mallorca), Santa Pola, Eilat, Canary Islands, Turkey, Algeria, and Colorada Chica (Argentina) showed ANI values >99.5%, but the conspecific MAGs form S’Avall (Mallorca) and Colorada Grande (Argentina) showed average ANI values of 98.2% ± 0.06% and 96.3% ± 0.11% to the remaining MAGs of the species. MAGs assigned to *Halovenus* sp_75_3 and *Salinirussus* sp_82_1 species had higher genetic similarity among coastal compared to inland sites, with a significant intra-genomovar ANIr distance–decay relationships among MAGs from coastal sites, accompanied by increasing SNP counts and a significant decrease in the proportion of synonymous SNPs with distance (Figs 4C and S10C). Yet, analysis of MAGs from inland sites showed no distance–decay relationships. In contrast, no significant correlations were detected between geographic distance and ANI, ANIr, SNP counts, or the proportion of synonymous SNPs for MAGs of *Halorubrum* sp_127_1, *Nanosalina* sp_219_1, *Halorubrum* sp_122_1, and *Haloferacaceae* sp_150_1 (Figs S7C and S10C). Again, for the five species for which MAGs were recovered from both coastal and inland sites, core-gene phylogenies showed that inland MAGs were the most divergent, forming distinct or separated phylogenetic branches (Fig. S10C).

### Ephemeral brines of the Chilean salterns of Lo Valdivia and the Mallorca coastal hypersaline pools (Cocos)

These two hypersaline environments are treated separately given their distinct microbial composition observed (Fig. 1C), which can be explained by their unique environmental dynamics. Similar to the Cocos, the Chilean brines of Lo Valdivia can be considered as ephemeral because the area is flooded annually with fresh water from a local river in winter and then refilled with seawater from the Pacific Ocean in spring, which is subsequently evaporated in small evaporation and crystallizer ponds to harvest salt for human consumption (Fig. S2). Therefore, flooding resets the succession of microbial communities each year. From the two Coco samples taken (ES_CO1 and ES_CO2; Table 1), biomass was similarly pigmented as other hypersaline sites (Fig. S2C). Mash distance (Fig. 1C) showed ES_CO1 to be the most dissimilar among all samples available, and ES_CO2 to be most similar to the two Chilean samples. We binned 15 MAGs from ES_CO1 and 18 MAGs from ES_CO2, which were affiliated with genera commonly found in hypersaline environments; another nine MAGs recovered from other sites were present in Cocos metagenomes based on read mapping but were not binned as MAGs (Table S6). Among all species detected from inland and coastal sites, 12 were exclusively present in the Cocos, and 42 were shared with other coastal and inland samples. Coco samples shared more species with coastal samples (14 ± 4 MAGs on average) than with inland samples (9 ± 7 MAGs on average; Fig. S11B). The greatest overlap of Coco in species was observed with the two Chilean pools, CL_LV1 and CL_VL2, sharing 21 and 25 species, respectively (Fig. S11A), and both sites also showed the highest similarity based on Mash distance (Fig. S11C and D).

## Discussion

Coastal samples showed higher genetic similarity than inland hypersaline sites with comparable global geographical distributions, and the closest sites displayed the highest similarities in both community structures and gene content. Coastal sites displayed elevated concentrations of magnesium and nitrogen salts (i.e. NO₃^−^ and NH₄^+^), and a greater heterogeneity in their ionic composition, compared to inland sites. The higher similarity among the coastal communities could be attributed to human controls on these solar salterns [32], or to higher environmental connectivity caused by efficient dispersal mechanisms [8, 9], including atmospheric transport [11–13], migratory birds [14, 15], or oceanic currents. In contrast, inland brines harbor more diverse microbial communities, which could be, at least partially, attributed to the lack of human intervention in many of them [32], and higher similarity in ionic composition. The presence of some microbes within ancient diapirs [31] may account for the higher species (or strain) diversity of inland sites, which would manifest as allopatry [60]. Additional abiotic and biotic variables not assessed by our study, including organic matter composition and concentration, temperature, and species interactions, could also play important roles in shaping microbial community assembly, and thus the diversity patterns revealed.

The coastal brines of Lo Valdivia and Cocos were outliers in our analyses by showing highly divergent microbial compositions. The most plausible explanation for these results is their ephemeral hypersaline conditions of these sites, which are annually disrupted during the cold/wet season, and thus often do not reach salt-saturation conditions and the usual composition of thalassohaline hypersaline environments [25]. It appears that microbial community re-establishment occurs after each seasonal transition, and may require prolonged periods of osmotic stability to reach the mature communities commonly found in other, less transient hypersaline sites [26, 32, 38, 68]. During the wet season, both systems become diluted by the influx of seawater, likely disrupting the previously established hypersaline microbial communities, which must subsequently reassemble during the following dry season. Consistently, we did not detect any trace of *Hqr. walsbyi* in the Lo Valdivia and Cocos samples, despite this species being the most abundant member in nearly all examined brines. A similar absence of *Hqr. walsbyi* has been reported in the brines of the Cahuil salterns [69], which are managed in a comparable manner to the Lo Valdivia and Cocos systems. It would be interesting to experimentally determine whether long-term stabilization of these ephemeral brines would result in convergent communities with stable hypersaline environments.

The collection of MAGs encompassed 284 species with aggregate relative abundance ranging from 50% to 90% of the total microbial communities, values that are generally higher than those reported for many complex marine and freshwater habitats, where the genome-resolved fraction recovered by sequencing is often substantially lower due to higher diversity [55, 70, 71]. About 115 of these species and ~70% of the OGs did not show any preference for coastal or inland environment, revealing a high taxonomic and functional redundancy between the two environments [25, 72, 73]. The high prevalence of the same taxa across the sampled sites and the steep spatial and temporal salinity gradients these sites represent [36, 37, 68] is most likely attributed, at least in part, to the high intraspecific microdiversity these species harbor that enables species adaptation and/or high resistance to changing conditions. These species often comprised multiple coexisting genomovars with distinct functional capacities [36, 37, 68] (note that we initially named these intra-species units as “ecotypes” [37] before redefining the concept of genomovar for them [33]). Most of these species are affiliated with a few archaeal and bacterial families, including *Haloferacaceae, Haloarculaceae, Nanosalinaceae,* and *Salinibacteraceae.*

The most abundant and ecologically successful species such as *Sal. ruber, Hns. pteroides*, and *Hqr. walsbyi* showed a distance–decay relationship with respect to their intraspecific genetic similarity. In these taxa, the number of shared genes with very high nucleotide identity among MAGs (≥99.6%) declined with increasing geographic distance of the samples that the MAGs were recovered from, at both short (0–400 km) and intermediate-to-long scales (500–19 000 km). This trend was supported by decreasing ANIr values, increasing SNP counts, and shifts towards an increasing proportion of non-synonymous (relative to synonymous) substitutions, indicating progressive genomic divergence across spatial scales. The increasing fraction of non-synonymous substitutions further points to functional differentiation, potentially reflecting local adaptation. Similar decoupling between nucleotide-level divergence and conserved metabolic potential has been attributed to stabilizing selection acting on core functions while permitting local adaptive divergence [74, 75], as shown for freshwater bacteria where these patterns can arise via different evolutionary processes, ranging from recombination-driven gene flow to more clonal populations undergoing gradual divergence [76]. Phylogenetic reconstructions corroborated these patterns, showing higher genetic cohesion among coastal MAGs compared to inland populations, which often affiliated as distinct clades, or exhibited longer branches which could indicate either accelerated evolution, or uncertain placement [77].

We also identified two *Haloquadratum* species displaying no distance–decay pattern. In both species, MAGs sharing ANI values >99.8%, considered to represent the same genomovar, were detected across geographically distant hypersaline systems worldwide. Although we cannot yet fully explain this finding, it suggests that the specific genomovars detected may be the best-adapted lineages of the species in these systems [38], outcompeting other genomovars with lower ecological fitness. The finding also indicates a highly efficient worldwide dispersal. Thus, our previous results also revealed that some taxa may show high dispersal efficiency, e.g. the species of the genus *Halococcus* are especially abundant in bird’s nostrils [14], which could facilitate dispersal via shorebird migration. Yet, other taxa such as the *Sal. ruber* and *Hqr. walsbyi* have distance–decay patterns indicative of dispersal limitation. Moreover, in addition to ecological and dispersal-based mechanisms, the remarkably low intra-species diversity observed in these *Haloquadratum* lineages may also reflect, at least in part, intrinsic evolutionary processes, such as reduced mutation rates and/or elevated homologous recombination. The occurrence of these highly clonal species may therefore result from a combination of strong ecological selection, dispersal processes, and lineage-specific evolutionary dynamics that constrain diversification. Quantifying the role of these processes in future studies will be important toward a more complete understanding of the biogeography patterns of hypersaline microbes, especially *Haloquadratum* species.

We also observed an additional diversity pattern characterizing several species with no clear correlation between ANI similarity and distance (Fig. 3). For this pattern, we do not have a clear hypothesis for the underlying mechanisms although it seems possible that incomplete sampling or low metagenome coverage could decrease statistical support for the resulting data could account for this pattern. It is also possible that other environmental parameters not measured by our study are responsible for the pattern, and thus a different sampling strategy would be required in the future to elucidate these environmental parameters and their effects (e.g. time-series samples from the sites and measurement of organic matter composition and/or biotic interactions, especially phage predation).

Altogether, the observed patterns point toward a general trend of allopatric divergence among the most prevalent species but also high dispersal, i.e. not accompanied by allopatric divergence for a few other taxa. However, the exact evolutionary mechanisms underlying these patterns remain difficult to resolve using MAG-based datasets alone. Microbial diversification may be driven by different processes, or their combinations, such as horizontal gene transfer (HGT) mediated by homologous or non-homologous recombination, point mutation followed by varied selection pressures and variation in mutation fixation rates, genome rearrangements, and other forces that can either promote or constrain population divergence. The contribution of these processes is challenging to assess based on MAGs that represent mosaic consensus sequences of coexisting genomovars [78] and thus cannot often distinguish—for example—between HGT and shifts in strain composition or abundance. Furthermore, analyses based on SNP patterns require highly resolved genome reconstruction at the strain level, which remains challenging for complex natural communities and short-read metagenomic data. Resolving these mechanisms and quantifying their relative importance for the diversity patterns observed will require future studies combining low-error long-read sequencing technologies (e.g. PacBio Sequel II), strain-resolved approaches, and extensive isolate and/or single-cell genome collections.

It seems that in all cases the larger genetic divergence among geographically distant sites can be explained by a combination of long-distance dispersal [79, 80] and evolutionary processes, although the relative contributions of these remains speculative at present as explained above. The pattern of overall higher genetic relatedness among coastal microbial communities can be explained by hydrological connectivity based on predictable currents carrying dormant halophiles over relatively small timescales. In contrast, the higher diversity harbored by inland sites may reflect historical contingencies [4]. Part of the historical context of the inland environments is their geological age as they represent either remnant water/brine bodies from larger seawater enclosures such as the Great Salt Lake dated from the Pleistocene (~30 000 to 12 000 years ago) or just brines resulting from the dissolution of ancient diapirs as old as the early Jurassic Period (201 to 145 million years ago, as it may be the case of Rio Maior [29]), carrying viable organisms from their origin diapirs. The current composition of all sites may also be influenced to some extent by the exogenous microbial immigration through aerial or animal transport [11–15], which has its own geographical and climatic constrains. Similar contrasts have been reported across a wide range of ecosystems previously [19]. It would be important to quantify the relative importance of these processes for the differences observed in the diversity patterns of coastal versus inland sites in the future. In any case, the high ubiquity of several species revealed by our data, especially for coastal sites and to a lesser extent for inland sites, indicates high connectivity, even at continental scales, which can facilitate gene flow and, as previously reported for pelagic freshwater bacteria [81], reduce or even prevent allopatric divergence. Yet, for several species, we also observed local adaptation (allopatric differentiation), presumably driven by the local conditions selecting for the observed genotypes, indicating that different species are subjected to different levels of dispersal and local adaptation.

### Sampling permits

Brine sampling in Spanish solar salterns was authorized by the Spanish Ministry of Ecological Transition (ESNC22, ESNC27). Great Salt Lake sampling was permitted by the State of Utah, and South African salterns by landowners. Sampling at Oum El Bouaghi-Sebkha Ezzemoul (Algeria) was approved by local authorities, and at Tuz Lake (Turkey) by site operators. In Argentina, permits were issued by the Dirección de Recursos Naturales (La Pampa). Fără Fund Lake (Romania) sampling was authorized by Ocna Sibiului city hall. All sampling complied with relevant permits and regulations.

## Supplementary Material

Supplementary_material_ycag165

## Data Availability

The genomes and metagenomes were deposited under the European Nucleotide Archive (ENA) accession code PRJEB45291 (Tables S3 and S8).

## Notes

Present address: Luis Miguel Rodriguez Rojas, Aalborg University, Department of Chemistry and Bioscience, 9220 Aalborg East, Denmark

## References

[1] Hellweger FL, van Sebille E, Fredrick ND. Biogeographic patterns in ocean microbes emerge in a neutral agent-based model. *Science* 2014;345:1346–9. 10.1126/science.1254421 25214628

[2] Whittaker KA, Rynearson TA. Evidence for environmental and ecological selection in a microbe with no geographic limits to gene flow. *Proc Natl Acad Sci USA* 2017;114:2651–6. 10.1073/pnas.1612346114 28209775 PMC5347567

[3] De Wit R, Bouvier T. ‘Everything is everywhere, but, the environment selects’; what did baas Becking and Beijerinck really say? *Environ Microbiol* 2006;8:755–8. 10.1111/j.1462-2920.2006.01017.x 16584487

[4] Martiny JBH, Bohannan BJM, Brown JH et al. Microbial biogeography: putting microorganisms on the map. *Nat Rev Microbiol* 2006;4:102–12. 10.1038/nrmicro1341 16415926

[5] Hanson CA, Fuhrman JA, Horner-Devine MC et al. Beyond biogeographic patterns: processes shaping the microbial landscape. *Nat Rev Microbiol* 2012;10:497–506. 10.1038/nrmicro2795 22580365

[6] Reno ML, Held NL, Fields CJ et al. Biogeography of the *Sulfolobus islandicus* pan-genome. *Proc Natl Acad Sci USA* 2009;106:8605–10. 10.1073/pnas.0808945106 19435847 PMC2689034

[7] Conrad RE, Rodriguez-R LM, Lindner BG et al. An ANIr-based methodology to determine if two sequence-discrete populations are identical and identify cosmopolitan prokaryotic populations. *ISME comm* 2026;6:ycag068. 10.1093/ismeco/ycag068 PMC13098170 42022012

[8] Lennon JT, Jones SE. Microbial seed banks: the ecological and evolutionary implications of dormancy. *Nat Rev Microbiol* 2011;9:119–30. 10.1038/nrmicro2504 21233850

[9] Hoehler TM, Jørgensen BB. Microbial life under extreme energy limitation. *Nat Rev Microbiol* 2013;11:83–94. 10.1038/nrmicro2939 23321532

[10] Álvarez-Noriega M, Burgess SC, Byers JE et al. Global biogeography of marine dispersal potential. *Nat Ecol Evol* 2020;4:1196–203. 10.1038/s41559-020-1238-y 32632257

[11] Smith DJ, Jaffe DA, Birmele MN et al. Free tropospheric transport of microorganisms from Asia to North America. *Microb Ecol* 2012;64:973–85. 10.1007/s00248-012-0088-9 22760734

[12] DeLeon-Rodriguez N, Lathem TL, Rodriguez-R LM et al. Microbiome of the upper troposphere: species composition and prevalence, effects of tropical storms, and atmospheric implications. *Proc Natl Acad Sci USA* 2013;110:2575–80. 10.1073/pnas.1212089110 23359712 PMC3574924

[13] Sarmiento-Vizcaíno A, Espadas J, Martín J et al. Atmospheric precipitations, hailstone and rainwater, as a novel source of Streptomyces producing bioactive natural products. *Front Microbiol* 2018;9:773. 10.3389/fmicb.2018.00773 29740412 PMC5924784

[14] Brito-Echeverría J, López-López A, Yarza P et al. Occurrence of *Halococcus* spp. in the nostrils salt glands of the seabird *Calonectris diomedea*. *Extremophiles* 2009;13:557–65. 10.1007/s00792-009-0238-2 19363644

[15] Yim KJ, Kwon J, Cha IT et al. Occurrence of viable, red-pigmented haloarchaea in the plumage of captive flamingoes. *Sci Rep* 2015;5:16425. 10.1038/srep16425 26553382 PMC4639753

[16] Custer GF, Bresciani L, Dini-Andreote F. Ecological and evolutionary implications of microbial dispersal. *Front Microbiol* 2022;13:855859. 10.3389/fmicb.2022.855859 35464980 PMC9019484

[17] Walters KE, Capocchi JK, Albright MBN et al. Routes and rates of bacterial dispersal impact surface soil microbiome composition and functioning. *ISME J* 2022;16:2295–304. 10.1038/s41396-022-01269-w 35778440 PMC9477824

[18] Fronhofer EA, Bonte D, Bestion E et al. Evolutionary ecology of dispersal in biodiverse spatially structured systems: what is old and what is new? *Philos Trans R Soc Lond Ser B Biol Sci* 2024;379:20230142. 10.1098/rstb.2023.0142 38913061 PMC11391287

[19] Louca S . The rates of global bacterial and archaeal dispersal. *ISME J* 2022;16:159–67. 10.1038/s41396-021-01069-8 34282284 PMC8692594

[20] Viver T, Conrad RE, Lucio M et al. Description of two cultivated and two uncultivated new *Salinibacter* species, one named following the rules of the bacteriological code: *Salinibacter grassmerensis* sp. nov.; and three named following the rules of the SeqCode: *Salinibacter pepae* sp. nov., *Salinibacter abyssi* sp. nov., and *Salinibacter pampae* sp. nov. *Syst Appl Microbiol* 2023;46:126416. 10.1016/j.syapm.2023.126416 36965279

[21] Casamayor EO, Massana R, Benlloch S et al. Changes in archaeal, bacterial and eukaryal assemblages along a salinity gradient by comparison of genetic fingerprinting methods in a multipond solar saltern. *Environ Microbiol* 2002;4:338–48. 10.1046/j.1462-2920.2002.00297.x 12071979

[22] Gomariz M, Martínez-García M, Santos F et al. From community approaches to single-cell genomics: the discovery of ubiquitous hyperhalophilic *Bacteroidetes* generalists. *ISME J* 2015;9:16–31. 10.1038/ismej.2014.95 24926861 PMC4274432

[23] Antón J, Rosselló-Mora R, Rodríguez-Valera F et al. Extremely halophilic bacteria in crystallizer ponds from solar salterns. *Appl Environ Microbiol* 2000;66:3052–7. 10.1128/AEM.66.7.3052-3057.2000 10877805 PMC92110

[24] Antón J, Oren A, Benlloch S et al. *Salinibacter ruber* gen. Nov., sp. nov., a novel, extremely halophilic member of the bacteria from saltern crystallizer ponds. *Int J Syst Evol Microbiol* 2002;52:485–91. 10.1099/00207713-52-2-485 11931160

[25] Mora-Ruiz MDR, Cifuentes A, Font-Verdera F et al. Biogeographical patterns of bacterial and archaeal communities from distant hypersaline environments. *Syst Appl Microbiol* 2018;41:139–50. 10.1016/j.syapm.2017.10.006 29352612

[26] Viver T, Orellana L, Gonzalez-Torres P et al. Genomic comparison between members of the *Salinibacteraceae* family, and description of a new species of *Salinibacter* (*Salinibacter altiplanensis* sp. nov.) isolated from high altitude hypersaline environments of the Argentinian Altiplano. *Syst Appl Microbiol* 2018;41:198–212. 10.1016/j.syapm.2017.12.004 29429564

[27] Frontera M . Salines de les Balears. Palma: Ed, Fundació Sa Nostra Caixa de Balears. 2005.

[28] Jaakkola ST, Ravantti JJ, Oksanen HM et al. Buried alive: microbes from ancient halite. *Trends Microbiol* 2016;24:148–60. 10.1016/j.tim.2015.12.002 26796472

[29] Huby TJC, Clarck DR, McKew BA et al. Extremely halophilic archaeal communities are resilient to short-term entombment in halite. *Environ Microbiol* 2021;23:3370–83. 10.1111/1462-2920.14913 31919959 PMC8359394

[30] Davison I, Barreto P. Deformation and sedimentation processes, and hydrocarbon accumulations on upturned salt diapir flanks in the Lusitanian Basin, Portugal. *Pet Geosci* 2021;27:2019–138. 10.1144/petgeo2019-138

[31] Ramos-Barbero MD, Viver T, Zabaleta A et al. Ancient saltern metagenomics: tracking changes in microbes and their viruses from the underground to the surface. *Environ Microbiol* 2021;23:3477–98. 10.1111/1462-2920.15630 34110059

[32] Liébana R, Viver T, Ramos-Barbero MD et al. Extremely halophilic brine community manipulation shows higher robustness of microbiomes inhabiting human-driven solar saltern than naturally driven lake. *MSystems* 2024;9:e00538–24. 10.1128/msystems.00538-24 38934645 PMC11324034

[33] Viver T, Conrad RE, Rodriguez-R LM et al. Towards estimating the number of strains that make up a natural bacterial population. *Nat Commun* 2024;15:544. 10.1038/s41467-023-44622-z 38228587 PMC10791622

[34] Rodriguez-R LM, Conrad RE, Viver T et al. An ANI gap within bacterial species that advances the definitions of intra-species units. *MBio* 2024;15:e02696–23. 10.1128/mbio.02696-23 38085031 PMC10790751

[35] Andrei AŞ, Robeson MS, Baricz A et al. Contrasting taxonomic stratification of microbial communities in two hypersaline meromictic lakes. *ISME J* 2015;9:2642–56. 10.1038/ismej.2015.60 25932617 PMC4817630

[36] Conrad RE, Viver T, Gago JF et al. Toward quantifying the adaptive role of bacterial pangenomes during environmental perturbations. *ISME J* 2022;16:1222–34. 10.1038/s41396-021-01149-9 34887548 PMC9039077

[37] Viver T, Conrad RE, Orellana LH et al. Distinct ecotypes within a natural haloarchaeal population enable adaptation to changing environmental conditions without causing population sweeps. *ISME J* 2021;15:1178–91. 10.1038/s41396-020-00842-5 33342997 PMC8182817

[38] Bustos-Caparros E, Viver T, Gago JF et al. Global dominance of *Haloquadratum walsbyi* by a single genomovar with distinct gene content and viral cohorts from close relatives. *ISME J* 2025;19:wraf165. 10.1093/ismejo/wraf165 40757481 PMC12400929

[39] Oren A . Bioenergetic aspects of halophilism. *Microbiol Mol Biol Rev* 1999;63:334–48. 10.1128/MMBR.63.2.334-348.1999 10357854 PMC98969

[40] Oksanen J, Kindt R, Legendre P et al. The vegan package. *Community Ecol Pack* 2007;10:631–7.

[41] Urdiain M, López-López A, Gonzalo C et al. Reclassification of *Rhodobium marinum* and *Rhodobium pfennigii* as *Afifella marina* gen. Nov. comb. nov. and *Afifella pfennigii* comb. nov., a new genus of photoheterotrophic *Alphaproteobacteria* and emended descriptions of *Rhodobium, Rhodobium orientis* and *Rhodobium gokarnense*. *Syst Appl Microbiol* 2008;31:339–51. 10.1016/j.syapm.2008.07.002 18774253

[42] Rodriguez-R LM, Konstantinidis KT. Nonpareil: a redundancy-based approach to assess the level of coverage in metagenomic datasets. *Bioinformatics* 2014;30:629–35. 10.1093/bioinformatics/btt584 24123672

[43] Ondov BD, Treangen TJ, Melsted P et al. Mash: fast genome and metagenome distance estimation using MinHash. *Genome Biol* 2016;17:1–14. 10.1186/s13059-016-0997-x 27323842 PMC4915045

[44] Peres-Neto PR, Jackson DA. How well do multivariate data sets match? The advantages of a procrustean superimposition approach over the Mantel test. *Oecologia* 2001;129:169–78. 10.1007/s004420100720 28547594

[45] Nurk S, Meleshko D, Korobeynikov A et al. metaSPAdes: a new versatile metagenomic assembler. *Genome Res* 2017;27:824–34. 10.1101/gr.213959.116 28298430 PMC5411777

[46] Li D, Luo R, Liu CM et al. MEGAHIT v1.0: a fast and scalable metagenome assembler driven by advanced methodologies and community practices. *Methods* 2016;102:3–11. 10.1016/j.ymeth.2016.02.020 27012178

[47] Hyatt D, Chen GL, Locascio PF et al. Prodigal: prokaryotic gene recognition and translation initiation site identification. *BMC Bioinformatics* 2010;11:119–30. 10.1186/1471-2105-11-119 20211023 PMC2848648

[48] Buchfink B, Reuter K, Drost HG. Sensitive protein alignments at tree-of-life scale using DIAMOND. *Nat Methods* 2021;18:366–8. 10.1038/s41592-021-01101-x 33828273 PMC8026399

[49] Rodriguez-R LM, Konstantinidis KT. The enveomics collection: a tool-box for specialized analyses of microbial genomes and metagenomes. *PeerJ* 2016;4:e1900v1.27123377

[50] Wu YW, Simmons BA, Singer SW. MaxBin 2.0: an automated binning algorithm to recover genomes from multiple metagenomic datasets. *Bioinformatics* 2016;32:605–7. 10.1093/bioinformatics/btv638 26515820

[51] Kang DD, Li F, Kirton E et al. MetaBAT 2: an adaptive binning algorithm for robust and efficient genome reconstruction from metagenome assemblies. *Peer J* 2019;7:e7359. 10.7717/peerj.7359 31388474 PMC6662567

[52] Chklovski A, Parks DH, Woodcroft BJ et al. CheckM2: a rapid, scalable and accurate tool for assessing microbial genome quality using machine learning. *Nat Methods* 2023;20:1203–12. 10.1038/s41592-023-01940-w 37500759

[53] Olm MR, Brown CT, Brooks B et al. dRep: a tool for fast and accurate genomic comparisons that enables improved genome recovery from metagenomes through de-replication. *ISME J* 2017;11:2864–8. 10.1038/ismej.2017.126 28742071 PMC5702732

[54] Jain C, Rodriguez-R LM, Phillippy AM et al. High throughput ANI analysis of 90K prokaryotic genomes reveals clear species boundaries. *Nat Commun* 2018;9:5114. 10.1038/s41467-018-07641-9 30504855 PMC6269478

[55] Rodriguez-R LM, Tsementzi D, Luo C et al. Iterative subtractive binning of freshwater chronoseries metagenomes identifies over 400 novel species and their ecologic preferences. *Environ Microbiol* 2020;22:3394–412. 10.1111/1462-2920.15112 32495495

[56] Chaumeil PA, Mussig AJ, Hugenholtz P et al. GTDB-Tk: a toolkit to classify genomes with the genome taxonomy database. *Bioinformatics* 2020;36:1925–7. 10.1093/bioinformatics/btz848 PMC7703759 31730192

[57] Quinlan AR, Hall IM. BEDTools: a flexible suite of utilities for comparing genomic features. *Bioinformatics* 2010;26:841–2. 10.1093/bioinformatics/btq033 20110278 PMC2832824

[58] Langmead B, Salzberg SL. Fast gapped-read alignment with Bowtie 2. *Nat Methods* 2012;9:357–9. 10.1038/nmeth.1923 22388286 PMC3322381

[59] Nayfach S, Pollard KS. Average genome size estimation improves comparative metagenomics and sheds light on the functional ecology of the human microbiome. *Genome Biol* 2015;16:51. 10.1186/s13059-015-0611-7 25853934 PMC4389708

[60] Conrad RE, Brink CE, Viver T et al. Microbial species and intraspecies units exist and are maintained by ecological cohesiveness coupled to high homologous recombination. *Nat Commun* 2024;15:9906. 10.1038/s41467-024-53787-0 39548060 PMC11568254

[61] Steinegger M, Söding J. MMseqs2 enables sensitive protein sequence searching for the analysis of massive data sets. *Nat Biotechnol* 2017;35:1026–8. 10.1038/nbt.3988 29035372

[62] Edgar RC . MUSCLE: multiple sequence alignment with high accuracy and high throughput. *Nucleic Acids Res* 2004;32:1792–7. 10.1093/nar/gkh340 15034147 PMC390337

[63] Ludwig W, Strunk O, Westram R et al. ARB: a software environment for sequence data. *Nucleic Acids Res* 2004;32:1363–71. 10.1093/nar/gkh293 14985472 PMC390282

[64] Darling AC, Mau B, Blattner FR et al. Mauve: multiple alignment of conserved genomic sequence with rearrangements. *Genome Res* 2004;14:1394–403. 10.1101/gr.2289704 15231754 PMC442156

[65] Boratyn GM, Thierry-Mieg J, Thierry-Mieg D et al. Magic-BLAST, an accurate RNA-seq aligner for long and short reads. *BMC Bioinformatics* 2019;20:405. 10.1186/s12859-019-2996-x 31345161 PMC6659269

[66] Richter M, Rosselló-Móra R. Shifting the genomic gold standard for the prokaryotic species definition. *Proc Natl Acad Sci USA* 2009;106:19126–31. 10.1073/pnas.0906412106 19855009 PMC2776425

[67] Bowers RM, Kyrpides NC, Stepanauskas R et al. Minimum information about a single amplified genome (MISAG) and a metagenome-assembled genome (MIMAG) of bacteria and archaea. *Nat Biotechnol* 2017;35:725–31. 10.1038/nbt.3893 28787424 PMC6436528

[68] Bustos-Caparros E, Viver T, Gago JF et al. Ecological success of extreme halophiles subjected to recurrent osmotic disturbances is primarily driven by congeneric species replacement. *ISME J* 2024;18:wrae215. 10.1093/ismejo/wrae215 39441989 PMC11544370

[69] Plominsky AM, Henríquez-Castillo C, Delherbe N et al. Distinctive archaeal composition of an artisanal crystallizer pond and functional insights into salt-saturated hypersaline environment adaptation. *Front Microbiol* 2018;9:1800. 10.3389/fmicb.2018.01800 30154761 PMC6102401

[70] Royo-Llonch M, Sánchez P, Ruiz-González C et al. Compendium of 530 metagenome-assembled bacterial and archaeal genomes from the polar Arctic Ocean. *Nat Microbiol* 2021;6:1561–74. 10.1038/s41564-021-00979-9 34782724

[71] Tully BJ, Graham ED, Heidelberg JF. The reconstruction of 2,631 draft metagenome-assembled genomes from the global oceans. *Sci Data* 2017;5:170203. 10.1038/sdata.2017.203 PMC5769542 29337314

[72] Papke RT, Zhaxybayeva O, Feil EJ et al. Searching for species in haloarchaea. *Proc Natl Acad Sci USA* 2007;104:14092–7. 10.1073/pnas.0706358104 17715057 PMC1955782

[73] Zhaxybayeva O, Stepanauskas R, Mohan NR et al. Cell sorting analysis of geographically separated hypersaline environments. *Extremophiles* 2013;17:265–75. 10.1007/s00792-013-0514-z 23358730

[74] Rosen MJ, Davison M, Bhaya D et al. Fine-scale diversity and extensive recombination in a quasisexual bacterial population occupying a broad niche. *Science* 2015;348:1019–23. 10.1126/science.aaa4456 26023139

[75] Vos M, Hesselman MC, Te Beek TA et al. Rates of lateral gene transfer in prokaryotes: high but why? *Trends Microbiol* 2015;23:598–605. 10.1016/j.tim.2015.07.006 26433693

[76] Hoetzinger M, Hahn MW, Andersson LY et al. Geographic population structure and distinct intra-population dynamics of globally abundant freshwater bacteria. *ISME J* 2024;18:wrae113. 10.1093/ismejo/wrae113 38959851 PMC11283720

[77] Bergsten J . A review of long-branch attraction. *Cladistics* 2005;21:163–93. 10.1111/j.1096-0031.2005.00059.x 34892859

[78] Konstantinidis KT, Rosselló-Móra R, Amann R. Uncultivated microbes in need of their own taxonomy. *ISME J* 2017;11:2399–406. 10.1038/ismej.2017.113 28731467 PMC5649169

[79] Poulain PM, Bussani A, Gerin R et al. Mediterranean surface currents measured with drifters: from basin to subinertial scales. *Oceanography* 2013;26:38–47. 10.5670/oceanog.2013.03

[80] Hernández-Carrasco I, Orfila A, Rossi V et al. Effect of small scale transport processes on phytoplankton distribution in coastal seas. *Sci Rep* 2018;8:8613. 10.1038/s41598-018-26857-9 29872142 PMC5988812

[81] Hoetzinger M, Pitt A, Huemer A et al. Continental-scale gene flow prevents allopatric divergence of pelagic freshwater bacteria. *Genome Biol Evol* 2021;13:evab019. 10.1093/gbe/evab019 33674852 PMC7936036

