# Supplementary material for: Metagenomics reveal unrestricted dispersal of extreme halophiles and higher connectivity among coastal vs. inland solar salterns and hypersaline lakes
Source: ISME Commun. 2026 Jun 13;6(1):ycag165. doi: 10.1093/ismeco/ycag165 (PMC13440315; doi:10.1093/ismeco/ycag165)
Supplement: Supplementary file 1 [file supplementary_material_ycag165.zip › Supplem_Viver_et_al_2025_ycag165.doc]

**Supplementary Material**

**Metagenomics reveal unrestricted dispersal of extreme halophiles and higher connectivity among coastal vs. inland solar salterns and hypersaline lakes**

**Tomeu Viver^1*^, Juan F. Gago^1^, Esteban Bustos-Caparros^1^, Borja Aldeguer-Riquelme^2,3^, Luis M. Rodriguez-R^4^, Ana S. Ramírez^5^, Luciana Albuquerque^6^, Souad Amiour^7^, Aharon Oren^8^, Mehmet Burcin Mutlu^9^, Stephanus N. Venter^10^, Bonnie K. Baxter^11^, María E. Llames^12^, Bernardo González^13^, Gustavo Rodríguez-Valdecantos^13^, Horia L. Banciu^14,15^, Matthew B. Stott^16^, Fernando Santos^3^, Brian P. Hedlund^17^, Josefa Antón^3^, Rudolf Amann^18^, Konstantinos T. Konstantinidis^2,19^, Ramon Rossello-Mora^1*^**

**Affiliations**

^1^ Marine Microbiology Group, Department of Animal and Microbial Biodiversity, Mediterranean Institute for Advanced Studies (IMEDEA, CSIC-UIB), Esporles, Spain.

^2^ School of Civil & Environmental Engineering, Georgia Institute of Technology, Atlanta, GA, USA.

^3^ Department of Physiology, Genetics and Microbiology, University of Alicante, 03690, San Vicent del Raspeig, Alicante, Spain.

^4^ Department of Microbiology, and Digital Science Center (DiSC), Universität of Innsbruck, Innsbruck, Austria.

^5^ Unidad de Epidemiología y Medicina Preventiva, IUSA, Facultad de Veterinaria, Universidad de Las Palmas de Gran Canaria, C/Trasmontaña s/n, Arucas, 35413, Canary Islands, Spain.

^6^ CNC-UC – Center for Neuroscience and Cell Biology and CIBB – Center for Innovative Biomedicine and Biotechnology, University of Coimbra, UC-Biotech, BiocantPark, Núcleo 04, Lote 8, 3060-197 Cantanhede, Portugal

^7^ Laboratory of Bioengineering, National Higher School of Biotechnology, Constantine, Algeria.

^8^ The Institute of Life Sciences, The Hebrew University of Jerusalem, The Edmond J. Safra Campus, 9190401 Jerusalem, Israel.

^9^ Department of Biology, Faculty of Science, Eskisehir Technical University, 26470 Eskisehir, Turkey.

^10^ Department of Biochemistry, Genetics and Microbiology, and Forestry and Agricultural Biotechnology Institute (FABI), University of Pretoria, Pretoria, South Africa.

^11^ Great Salt Lake Institute, Westminster University, Salt Lake City, UT, 84105, USA.

^12^ Laboratorio de Ecología Microbiana Ambiental, Instituto Tecnológico de Chascomús (CONICET-UNSAM), Escuela de Bio y Nanotecnologías (UNSAM), Buenos Aires, Argentina.

^13^ Facultad de Ingeniería y Ciencias, Universidad Adolfo Ibáñez–Center for Applied Ecology and Sustainability, Santiago de Chile, Chile

^14^ Department of Molecular Biology and Biotechnology, Faculty of Biology and Geology, Babeș-Bolyai University, Cluj‑Napoca, Romania

^15^ Emil G. Racoviță Institute, Babeș-Bolyai University, Cluj‑Napoca, Romania

^16^ School of Biological Sciences, University of Canterbury, Christchurch, New Zealand

^17^ School of Life Sciences, University of Nevada, Las Vegas, Nevada 89154-4004, USA

^18^ Department of Molecular Ecology, Max Planck Institute for Marine Microbiology, Bremen, Germany.

^19^ Ocean Science & Engineering, School of Biological Sciences, Georgia Institute of Technology, Atlanta, GA, USA.

**Corresponding author:**

Ramon Rossello-Mora and Tomeu Viver

Marine Microbiology Group

Department of Animal and Microbial Biodiversity

Mediterranean Institute for Advanced Studies (IMEDEA, CSIC-UIB)

E-07190, Esporles

Spain

Tel: +34 971 611 827

Email: [ramon@imedea.uib-csic.es](mailto:ramon@imedea.uib-csic.es) and [tviver@imedea.uib-csic.es](mailto:tviver@imedea.uib-csic.es)

**Key Words:** Halophiles, distance decay patterns, genomics, metagenomics, ANI, biogeography

**Supplementary Text S1: MATERIAL AND METHODS**

**Samples: origin and processing**

In year 2018, an international research consortium formed the Halophile Sequencing Project (HSP), involving 19 researchers from 11 countries worldwide. The HSP team collected 25 samples from 11 countries (Table 1). All samples were thalassohaline, i.e. with a direct (seawater) or indirect (dissolution of diapiric rocks) ocean water origin. For each sample, at least 1 L of brine was collected and used for the current metagenomic study. All samples were collected between September 2018 and January 2020, and were close to saturation levels for NaCl (25% to 37% total salts) at the time of sampling.

**Ionic composition of the samples in study**

To measure the ionic composition and concentration, samples were filtered through 0.22 μm hydrophilic PTFE filters, and the concentrations of fluoride (F^-^), chloride (Cl^-^), bromide (Br^-^), nitrate (NO_3_^-^), sulfate (SO_4_^2-^), phosphate (PO_4_^3-^), sodium (Na^+^), lithium (Li^+^), potassium (K^+^), ammonium (NH_4_^+^), magnesium (Mg^2+^), and calcium (Ca^2+^) were quantified by ion chromatography using a Metrohm, 850 ProfIC AnCat — MCS system, by the Technical Research Services of Alicante University (Spain). The salinities of the samples were measured using a Sper Scientific Salt Refractometer (model number 300006, Arizona, USA).

To investigate differences in ionic composition among samples, we conducted multivariate analyses in R (v4.2.3) using the vegan package [39] package. Similarity among samples based on ionic composition was assessed using Principal Component Analysis (PCA) on scaled data and Non-Metric Multidimensional Scaling (NMDS) based on Bray–Curtis dissimilarity. To evaluate statistically significant differences in ionic composition between sample groups, we applied permutational multivariate analysis of variance (PERMANOVA, adonis2) and analysis of similarities (ANOSIM, anosim), both performed on Bray–Curtis distance matrices.

**Metagenome analyses**

DNA extraction from environmental samples was performed as detailed in Urdiain and colleagues [40]. Metagenomic samples were sequenced using an Illumina NextSeq500 instrument (2 x 150 bp, paired-end reads). Bbduk within the Bbtools package (http://bbtools.jgi.doe.gov/) was used to trim the paired-end reads with the following options: ktrim=r, k=28, mink=12, hdist=1, tbo=t, tpe=t, qtrim=rl, trimq=20 and minlength=100. Abundance-weighted average coverage and alpha-diversity indices of the metagenomes were estimated using Nonpareil v3.303 [41]. Beta-diversity analysis was determined using Mash distance v2.2.2 [42] and visualized in an NMDS plot using vegan v2.6 [39]. The correlation between Mash distances with geographic distance and ion concentration was assessed using Procrustes analysis [43].

Trimmed reads were assembled with the SPAdes v3.13.1 [44] assembler using metaSPAdes mode and MEGAHIT v1.2.9 [45] with default parameters. Gene prediction from assembled contigs with > 500 bps length was conducted using Prodigal v2.6.3 [46]. All predicted proteins from all metagenomes were compared using an all-versus-all DIAMOND blast tool v0.9.31 [47] and shared reciprocal best matches in all pairwise genome comparisons were identified using *rbm.rb* script [48] using a 50% sequence identity cut-off over 50% or more of the query sequence length. The orthologous group (OG) proteins were identified using *ogs.mcl.rb* script [48]. The frequency of each OG within the two sample sets (coastal vs. inland) was estimated using the *Table.prefScore.R* script [48]. Briefly, this approach compares the observed and expected frequency of each OG across metagenomes, normalizing for both the presence frequency of the OG and the overall abundance of OGs per sample. The ratio between observed and expected frequencies (maintaining the sign of the observed bias) defines the preference score. The representative sequence from each OG was annotated using the COG classifier v1.0.5 tool (https://github.com/moshi4/COGclassifier). Amino acid identity (AAI)-based similarity was visualized using NMDS plots generated with the vegan R package, applying a range of identity thresholds from 50% to 90%.

**MAG reconstruction and relative abundance**

From the assembled files, contigs larger than 2,000 bps were binned using MaxBin v2.1.1 software [49] and MetaBAT v2 software [50]. Completeness and contamination values from MAGs were calculated using the CheckM2 tool v0.1.3 [51]. MAGs with completeness > 50% and contamination <10% were selected for further analysis. The resulting bins from each metagenome were dereplicated with dRep v3.4.0 [52], selecting the highest-quality MAG for each species-level cluster based on a 96% ANI threshold. Subsequently, to obtain one representative MAG per species across all metagenomes, the selected MAGs were re-clustered with dRep at 96% ANI (using the FastANI algorithm [53]) and a minimum alignment overlap of 20% [54]. A representative MAG from each species was selected manually considering the higher completeness and lower contamination values. The phylogenetic placement of the representative MAGs was analysed using the GTDB-Tk v2.4 tool with the GTDB Release R220 [55]. Species abundance was estimated using the representative selected MAG as a reference. For each metagenomic dataset, the MAG sequencing depth was estimated per position (Bowtie2; bedtools) [56,57] and truncated to the central 80% values (i.e. removing the top and bottom 10% positions by depth) using the *BedGraph.tad.rb* script [48], a metric hereafter termed TAD80 (truncated average sequencing depth). Abundance was estimated as TAD80 normalized by the genome equivalents of the metagenomic dataset determined using the MicrobeCensus tool v1.1.0 [58]. A species was considered to be present in a sample if the TAD80 was non-zero and showing a sequencing breadth ≥ 20% (i.e. at least 20% of the genome covered by metagenomic reads). In order to estimate the frequency of a species in a given set of samples (i.e. accounting for the geographic distance or environmental characteristics) compared to the rest, we used the *Table.prefScore.R* script [48]. This approach compares the observed and expected frequency of each MAG across metagenomes, normalizing for both the presence frequency of the MAG and the overall abundance of MAGs per sample. The ratio between observed and expected frequencies (maintaining the sign of the observed bias) defines the preference score.

**Intra-species genome analysis**

The average nucleotide identity (ANI) and the fraction of the genome shared between MAGs belonging to the same species were determined based on BLAST searches using *ani.rb* script [48]. Intra-species pangenome analysis was conducted following the methodology described by Conrad and colleagues [59]. Briefly, predicted genes were clustered into orthologous groups (OGs) at 90% nucleotide identity across at least 70% of the query sequence length using MMseqs2 v13.45111 [60], according to the guidelines provided in the pangenome workflow manual reported in https://github.com/rotheconrad/F100_Prok_Recombination. Single-copy core genes present in all MAGs of the same species were individually aligned using Muscle v3.8.31 [61] and concatenated with the script *Aln.cat.rb* [48]. Phylogenetic analyses were performed using RAxML implemented in ARB v6.0.6 [62]. The number of Single Nucleotide Polymorphisms (SNPs) between MAGs pair of the same species were predicted with Mauve [63], and synonymous versus non-synonymous SNPs were identified using a custom Python pipeline. Briefly, CDSs from a reference genome were mapped to homologous regions via BLAST, aligned with strand correction, and truncated to full codons. Codon changes were classified as synonymous if the amino acid was unchanged or non-synonymous if altered, and counts were summarized per CDS and aggregated genome-wide. Average Nucleotide Identity of reads (ANIr) was calculated by mapping metagenomic reads to reference MAGs using Magic-BLAST+ v2.12.0 in BLASTn mode [64], retaining reads with > 95% identity and averaging across all selected reads.

**Figure S1.** Geographical distribution of the hypersaline samples collected in this study. All samples were collected between September 2018 and August 2019. Coastal or inland sites are marked with red or blue star, respectively. The distances given refer to Mallorca as a reference (geographic distances between pair of location have been reported in Table S9). Background colors indicate the geographic distance between samples, classified as short (0–400 km; green), intermediate (400–5,000 km; red), and long distances (5,000–20,000 km; blue).

**Figure S2.** Sampling of hypersaline brines from Coco pools (Mallorca Island, Spain) and Lo Valdivia solar salterns (Chile). (A) Geographic location of Coco pools on the southern rocky coast of Mallorca Island; (B, D, E) field sampling of brine from Coco 1 and Coco 2 pools; (C) laboratory-processed brine samples in bottles; (F) brine pellets obtained after centrifugation, used for DNA extraction and metagenomic sequencing. (G, H) Lo Valdivia salterns during the winter season, when ponds are flooded by seawater intrusions from the nearby river during high tide; (I, J) Lo Valdivia salterns in summer, showing the formation of crystallizer ponds by seawater evaporation and concentration of brines.

**Figure S3.** Metagenomic alpha diversity index based on Nonpareil (*N_d_*) (A) for each individual sample, and (B) boxplot differentiated by coastal solar saltern and inland hypersaline lake origin.

**Figure S4.** Preference scores of the protein orthologous groups (OGs) predicted from the assembled metagenomes. (A) OGs with preference scores >9 indicate a higher prevalence in coastal salterns (red), while scores <9 indicate a greater prevalence in inland samples (blue). (B) Each point represents the presence or absence of OGs across the samples included in the study. (C) Metabolic classification of the OGs based on COG functional categories (see legend), differentiating between coastal (red bars), inland (blue bars), or no specific environmental preference (grey bars).

**Figure S5.** Total abundance of species detected by MAGs in each metagenome. Bars are color-coded by sample origin: red for coastal, blue for inland, purple for Lo Valdivia, and yellow for Cocó samples.

**Figure S6.** Relative abundance of the 21 species recovered from MAGs used for intraspecies comparisons. Colors indicate sample origin: coastal sites (red), inland sites (blue), Chilean coastal salterns of Lo Valdivia (pink), and Coco samples (yellow).

**Figure S7.** Intra-species average nucleotide identity (ANI, y-axes) compared to geographic distances (x-axis). The comparisons are based on pairwise ANI analyses between MAGs assigned to the same species. (A) Species showing an ANI decrease with increasing geographic distance. (B) Highly conserved species with ANI values >99%. (C) Species with no correlation between ANI and geographic distance.

**Figure S8.** Intra-species average nucleotide identity (ANI, y-axes) compared to geographic distances (x-axis), considering just the intra-species core genome for ANI value calculations. (A) Species showing an ANI decrease with increasing geographic distance. (B) Highly conserved species with ANI values >99. (C) Species with no correlation between ANI and geographic distance.

**Figure S9.** **Intraspecies variation in core genes similarity across geographic distances**. To assess the impact of geographic distance on genomic divergence within species, we compared the sequence similarity of shared core genes between MAGs recovered from different sampling sites and to the same species. Pairwise comparisons were grouped by geographic distance into short-range (0–400 km; red), intermediate-range (401–5000 km; green), and long-range (5001–22,000 km; blue) categories. Each plot displays the average distribution of core gene similarity values for all pairwise comparisons within each distance group. Each line represents the distribution of core-gene similarity values binned into 0.2% intervals. The four panels are organized according to Figure 3: (A) species showing an ANI decrease with increasing geographic distance; (B) highly conserved species with ANI values >99; (C) species with no correlation between ANI and geographic distance. The table within each panel reports the percentage of core genes exhibiting >99.5% or <99% sequence similarity across the different geographic distance ranges.

**Figure S10. Comparison of the relationship between ANI and geographic distance with patterns observed in the core-gene phylogenetic tree, ANIr, number of SNPs, and the proportion of synonymous SNPs across increasing geographic distances.** This figure presents the full set of species analyzed, whereas Figure 4 shows a subset of representative examples for each pattern to enhance clarity and reduce complexity in the main text. A) Species showing a decrease in ANI (y-axes) with increasing geographic distance (x-axes). (B) Highly conserved species with ANI values >99. (C) Species with no correlation between ANI and geographic distance. The outgroups in the phylogenetic trees, highlighted in black, were selected from MAGs of closely related species or genera within our collection. In the trees, MAGs from coastal sites are labeled in red, while those from inland sites are labeled in blue.

**Figure S11.** Number of MAGs shared between Coco with coastal, inland and Chilean (Lo Valdivia) metagenomes. (A) Barplot indicating the number of MAGs shared between coco and all the other samples. (B) Boxplot differentiated by coastal solar saltern, inland hypersaline lakes and Lo Valdivia samples. (C) Boxplot comparing the Mash distance dissimilarity values between the Coco samples to coastal, inland or the Chilean samples. (D) Boxplot comparing the Mash distance dissimilarity between the Chilean samples to coastal, inland or the Coco samples.
